# Imaging biomarkers in cardiac CT: moving beyond simple coronary anatomical assessment

**DOI:** 10.1007/s11547-024-01771-5

**Published:** 2024-02-06

**Authors:** Giulia Cundari, Livia Marchitelli, Giacomo Pambianchi, Federica Catapano, Luca Conia, Giuseppe Stancanelli, Carlo Catalano, Nicola Galea

**Affiliations:** 1 Department of Radiological, Oncological and Pathological Sciences, Sapienza University of Rome, Viale Regina Elena 324, 00161 Rome, Italy; 2 Department of Biomedical Sciences, Humanitas University, Via Rita Levi Montalcini, 4, Pieve Emanuele, 20090 Milano, Italy; 3 Humanitas Research Hospital IRCCS, Via Alessandro Manzoni, 56, Rozzano, 20089 Milano, Italy

**Keywords:** Cardiac computed tomography angiography, Imaging biomarkers, Coronary artery disease, Myocardial tissue characterization, Fractional flow reserve, Myocardial perfusion

## Abstract

Cardiac computed tomography angiography (CCTA) is considered the standard non-invasive tool to rule-out obstructive coronary artery disease (CAD). Moreover, several imaging biomarkers have been developed on cardiac-CT imaging to assess global CAD severity and atherosclerotic burden, including coronary calcium scoring, the segment involvement score, segment stenosis score and the Leaman-score. Myocardial perfusion imaging enables the diagnosis of myocardial ischemia and microvascular damage, and the CT-based fractional flow reserve quantification allows to evaluate non-invasively hemodynamic impact of the coronary stenosis. The texture and density of the epicardial and perivascular adipose tissue, the hypodense plaque burden, the radiomic phenotyping of coronary plaques or the fat radiomic profile are novel CT imaging features emerging as biomarkers of inflammation and plaque instability, which may implement the risk stratification strategies. The ability to perform myocardial tissue characterization by extracellular volume fraction and radiomic features appears promising in predicting arrhythmogenic risk and cardiovascular events. New imaging biomarkers are expanding the potential of cardiac CT for phenotyping the individual profile of CAD involvement and opening new frontiers for the practice of more personalized medicine.

## Introduction

Ischemic heart disease (IHD) is the main cause of mortality in the world, responsible for around 16% of the total deaths [1]. Current prevention strategies rely on the risk stratification, acting with pharmacological treatments or lifestyle habits, and early detection of obstructive coronary artery disease (CAD). Clinical scores systems, electrocardiogram and echocardiogram demonstrated low sensitivity and specificity for the early diagnosis of CAD and prediction of major cardiovascular events (MACE) risk [2, 3]. In the last decades, cardiac computed tomography angiography (CCTA) has gained a preeminent role in the evaluation of symptomatic patients with suspected CAD, thanks to its high diagnostic accuracy and high negative predictive value. According to recent guidelines, CCTA can be considered the exam of choice to rule out obstructive CAD in patients with chronic cardiac symptoms and low clinical pre-test likelihood of disease [4–6], and in patients with acute chest pain with low-to-intermediate pre-test probability [7, 8]. However, the pure anatomical assessment of coronary arteries obtained with CCTA, does not provide functional information on lesion-specific ischemia. Recent developments in hardware and software technology, particularly with the introduction of artificial intelligence (AI) tools, are improving image quality of CCTA, increasing the detectable features of CAD (i.e., evaluation and quantification of coronary stenosis, plaque characterization, assessment of myocardial ischemia) and expanding the prognostic role of CCTA with machine-learning (ML) algorithms [9, 10]. Moreover, the use of dual energy CT (DECT) and the recent introduction of photon-counting detector scanners (PCD-CT) enabled the acquisition of ultra-high resolution images, with spectral information obtained along with each CT scan (material decomposition) and the reduction of blooming or movement artifacts together with the elimination of electronic noise [11]. The introduction of sophisticated postprocessing tools gave rise to innovative imaging biomarkers, which are, by definition, “parameters that can be measured and that may influence or predict the incidence of outcome of diseases” [12]. This new quantitative approach could improve management of patients and clinical decision making, moving toward a progressively targeted and personalized medicine. This review will focus on emerging CT imaging biomarkers, which are expanding the role of cardiac CT in individual phenotyping of CAD involvement, improving assessment of coronary stenosis and risk stratification, and characterizing myocardial tissue abnormalities.

## Atherosclerotic burden

**Fig. 1:**
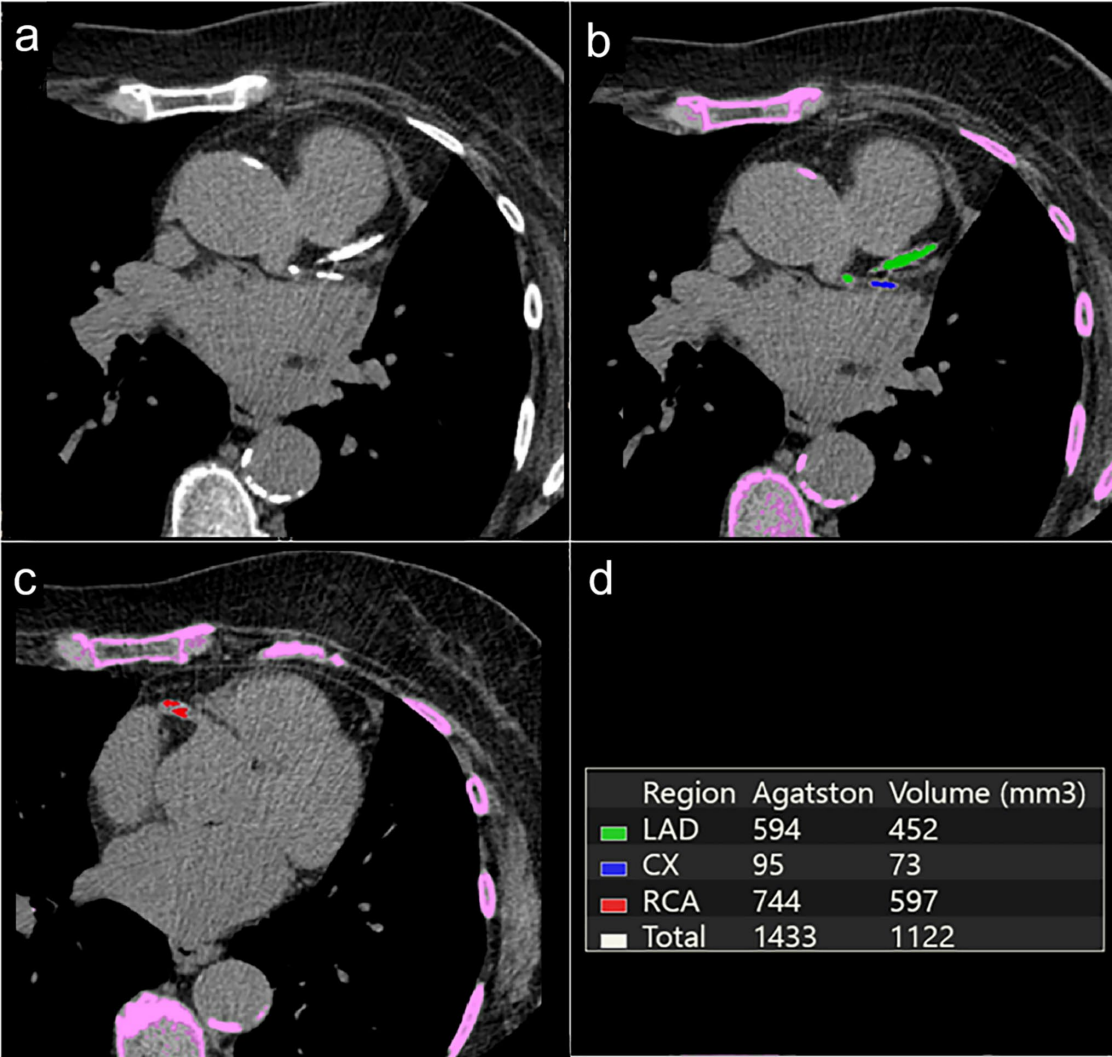
Coronary Calcium Scoring. Non-enhanced CT image showing coronary calcifications on proximal LAD and CX (**a**) followed by color-coded trans-axial images that highlights moderate calcifications on proximal LAD, CX (**b**) and RCA (**c**). Agatston Score is calculated to quantify the extent of coronary calcium (**d**). CT, computed tomography; LAD, Left Anterior Descending artery, CX, Circumflex Artery; RCA, Right Coronary Artery

Going beyond the detection of coronary stenosis, an increasing role of CCTA is represented by the assessment of atherosclerotic burden (even in patients with non-obstructive CAD). Several CCTA scores have been developed to guide risk stratification and clinical decision-making. The Agatston score (AS) quantifies the calcium load within the coronary arteries and is globally recognized as a robust test to classify the degree of CAD e to implement cardiovascular risk stratification [13]. The coronary artery calcium (CAC) scoring, indeed, represents a class-IIa recommendation test in patients with a borderline/intermediate risk, helping in management and therapeutic tailoring [14]. CAC quantification is performed using prospective ECG-gated unenhanced CT scan [15]. The standard image analysis is based on the segmentation of any structure with density ≥ 130 Hounsfield Units (HU) and having an area ≥ of 1 mm^2^ along coronary walls, as calcified focus plaque. In each segmented calcified plaque, a density score of 1–4 is assigned to each focus based on peak density (130–199 HU, 200–299 HU, 300–399 HU and ≥ 400 HU, respectively). The total AS is the result of the sum the scores of every coronary artery calcified focus [16] (Fig. 1).

CAC may also be measured in terms of Volume and Mass score, which measures the absolute real volume and mass of coronary calcium [15, 17, 18]. CAC quantification was found to be an excellent index of atherosclerotic plaque burden [19], showing an association between coronary calcification area, plaque volume and extent of atherosclerosis in vivo [20, 21].

Some AS cut-offs (0 = very low, 1–100 = low, 101–400 = intermediate, > 400 = high, > 1000 very high) were also proposed to differentiate in risk categories, with 10-years event rate of 22.5–28.6% and 37% in high and very high category, respectively [22]. In a recent metanalysis focusing on the role of CAC score in patients with stable or acute chest pain, the absence of CAC was associated with a very low prevalence of obstructive CAD and low risk of MACE. These results suggest the CAC score may play a role in identifying patients with stable and acute chest pain who can safely avoid additional downstream testing [23]. The accuracy of CAC quantification and classification has gradually improved over the years, in particular the CAC based on spectral data acquired with DECT [24] and new PCD-CT system allows for more accurate CAC volume estimation [25]. Both DECT and PCD-CT enable the quantification of CAC score on virtual non-contrast images (VNC), with a good agreement in assessing CAC risk categories compared to true non-contrast images [24, 26] and with a substantial increase in spatial resolution in PCD-CT. These techniques also decrease the radiation dose by eliminating the requirement for native scans typically used in standard CAC assessment.

Atherosclerotic burden may be also assessed on CCTA images. Segment Involvement Scoring (SIS) is a simple and reliable semiquantitative tool that quantifies CAD burden on CCTA (regardless of the stenosis degree), with a score ranging from 0 to 16, indicating the total number of coronary segments affected by atherosclerotic plaques, irrespective to the stenosis degree caused [27, 28]

**Fig. 2:**
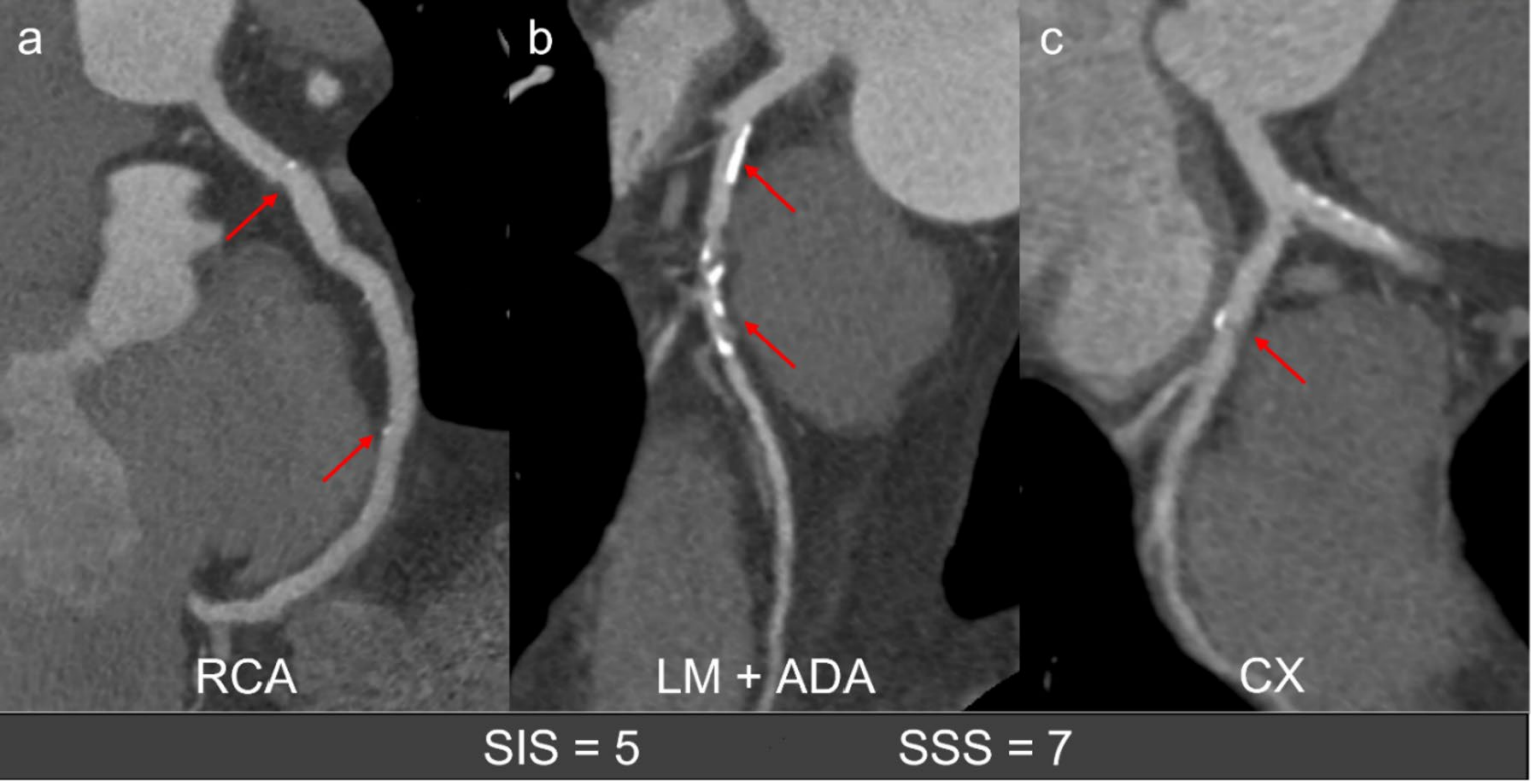
Segment Involvement Score (SIS) and Segment Stenosis Score (SSS). Curved multiplanar reformatted images show a total score of 5 for SIS and a total score of 7 for SSS. Minimal stenosis both on proximal and mid RCA (1 point each, for SIS and SSS; **a** mild stenosis on proximal LAD (1 point for SIS and SSS; **b** severe stenosis on mid LAD (1 point for SIS and 3 points for SSS; **b** and mild stenosis on proximal CX (1 point for SIS and SSS; **c**. CX, Circumflex Artery; LAD, Left Anterior Descending artery; LM, Left Main; RCA, Right Coronary Artery

SIS considers also the non-calcified plaques, which may not be detected by CAC scoring scan, implementing the prognostic stratification also at early stages of CAD [28]. Based on the number of segments with disease, extent of CAD may be classified as non-extensive (≤ 4 segments) or extensive (> 4 segments). Bittencourt et al. [29] demonstrated that among patients with nonobstructive CAD (stenosis < 50%), those with extensive plaque experienced a higher rate of cardiovascular death or myocardial infarction (hazard ratio—HR, 3.1, 95% confidence interval, 1.5–6.4), than those who have non-extensive disease (HR: 1.2, 95% CI 0.7–2.4) [29]. SIS can also be combined with patients’ age, in the “%SIS/age score”, which adjusts SIS to the number of evaluable segments and normalizes it to patient age, with an incremental prognostic value for MACE over traditional risk factors, Agatston score and conventional CAD assessment [27]. A further evolution of the CAD categorization system was the segment stenosis score (SSS), which is generated by the sum of the scores assigned for each coronary segment, based on the degree of the vessel lumen stenosis from 0 (absence of plaques) to 3 (severe stenosis), resulting in a total score ranging from 0 to 48. The SSS showed to be an independent predictor of all-cause mortality despite the patient’s age [30] (Fig. 2).

Coronary artery disease-reporting and data system (CAD-RADS) [31] aims to improve the accuracy of diagnosing and managing CAD patients. The updated 2022 CAD-RADS 2.0 classification [32] includes new elements such as plaque burden and ischemia evaluation, enabling the integration of CT-FFR or myocardial CT perfusion (CTP) data; moreover it introduces modifiers like coronary stents, high-risk plaque features, ischemia test results, and the "P" designation to classify plaque severity, based on CAC, SIS, and Visual scoring for plaque categorization [32]. Using a scale from 0 to 5, it grades coronary artery stenosis observed in CCTA images. Further investigation or hospital admission is required only for CAD-RADS categories 3, 4, and 5, whereas Invasive coronary angiography (ICA) is suggested for CAD-RADS 4 and 5 due to a likely/very likely assessment of obstructive coronary artery disease.

CT-adapted Leaman score (CT-LeSc), is based on three sets of weighting factors using a 18-segment coronary model: localization of plaques, accounting for the coronary dominance; the type of plaque, with a multiplication factor of 1 for calcified plaques and of 1.5 for noncalcified and mixed plaque; the degree of stenosis, with a multiplication factor of 0.615 for nonobstructive (< 50% stenosis) and of 1 for obstructive (≥ 50% stenosis) lesions. The final score is calculated as the sum of the partial CT-LeSc of all evaluable coronary segments [33]. CT-LeSc showed a significant association with some traditional demographic and clinical risk factors as well as scores for pretest CAD probability and cardiovascular risk [33, 34] and demonstrated to be an independent long-term predictor of MACEs. Patients with nonobstructive CAD with a significant atherosclerotic load (CT-LeSc > 5) exhibited event-free survival compared to patients with obstructive CAD.

The Leiden CCTA risk score is a comprehensive semiquantitative evaluation of the coronary segments, that uses different weight factors, such as the plaque location (0–6 points), the severity of the stenosis (1–1.4 points) and the composition (1–1.3 points) of coronary plaques; the score is the result of addition of each individual segment scores, which are obtained by the multiplication of these three factors [35]. It demonstrated to be able to predict major adverse cardiac events (MACEs) in both diabetic and non-diabetic patients, with suspected CAD [36].

Major limitation to the broad routine use of the quantitative or semiquantitative assessment of coronary atherosclerotic burden is the long-time image analysis and scoring calculation. AI algorithms appear promising in speeding up this process, warranting greater reproducibility and fast labeling of these data. ML and deep learning (DL) based techniques are improving image segmentation, quantification of plaques extent, stenosis assessment, identification of culprit coronary lesions and calculation of composite scores. The identification of the coronary artery stenosis severity is the most fundamental application of ML analysis (i.e., it can automatically identify coronary obstructive lesions, or classify minor coronary plaques) [37]. In this regard, Sandstedt et al. compared AI-based automatic CAC score evaluation on non-contrast CT images to semiautomatic software in 315 patients, finding an excellent correlation and agreement for three CAC scores (AS, volume score and mass score) and the number of calcified lesions (*p* = 0.935, 0.932, 0.934) [38].

## High-risk coronary plaque features

**Fig. 3:**
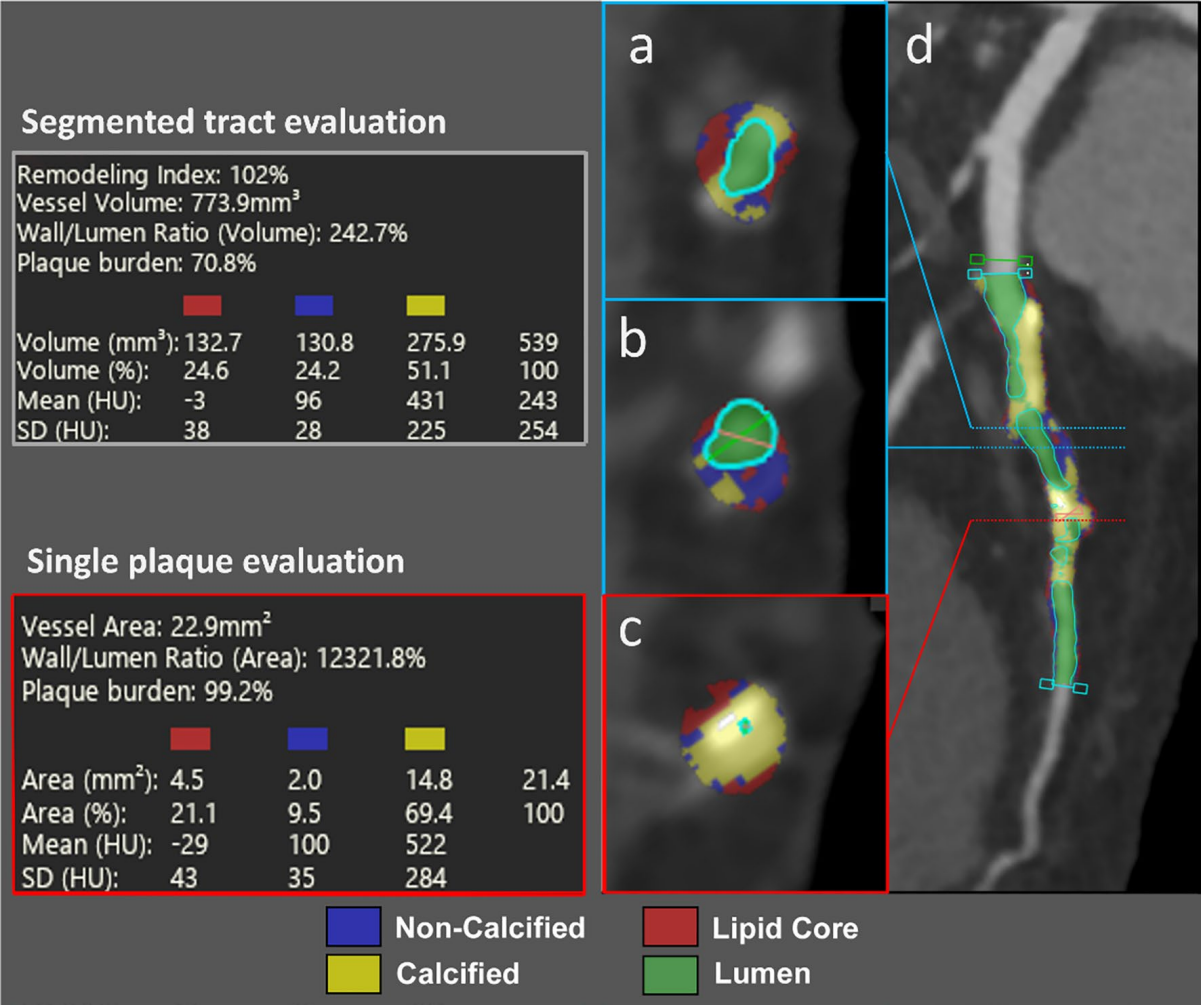
Curved multiplanar reformatted image of LAD (**d**) shows multiple mixed plaques (**a**–**c**) involving proximal and mid segments. The specific components of the plaques (non-calcified, calcified and lipid core) are analyzed and quantified automatically with a dedicated software, according to their density (on the left). LAD, Left Anterior Descending artery

CCTA may characterize the coronary plaque identifying high risk plaque features [39–41], which include:

- positive remodeling: defined as a remodeling index (RI) ≥ 1.1 [39, 42, 43], obtained by dividing the largest stenosis vessel cross-sectional area/diameter by the average cross-sectional area/diameter of the proximal and distal reference segments [44];
- intraplaque low attenuation regions: defined as mean attenuation < 30 HU in at least three regions of interest within the plaque [44] (Fig. 3);
- spotty calcifications: calcifications with more than 130 HU and a diameter < 3 mm encircled by non-calcified components [45, 46];
- napkin-ring sign: a low-attenuation core surrounded by annular high-attenuation plaque tissue [47].

Although CAD severity proved to be associated with the incidence of CV death and/or myocardial infarction (MI) [48], the PROMISE trial demonstrated that more than a half of MACE occurs in patient without coronary obstruction, suggesting that other factors must be taken into account [49]. Recent studies found an increased risk of MACE when high-risk plaques were detected on CCTA [39, 50, 51], regardless of CV risk factors and significant CAD, both for patients with stable angina and for patients admitted to the emergency department [40, 52]. Moreover, the detection of high-risk plaques can be useful in identifying significant lesions, as shown in the study from von Knebel Doeberitz et al. [53]: they found that lesion length, non-calcified plaque volume, RI, and “napkin-ring sign” were significant predictors for lesion-specific ischemia, as assessed by invasive fractional flow reserve (FFR).

Nevertheless, manual plaque quantification is time-consuming; consequently, semi-automatic plaque assessment using dedicated software has been recently introduced [54]. Semi-automated tools showed a high reproducibility (in both intra- and inter-observer comparisons) for CCTA geometrical measurements (such as lumen and vessel area and plaque burden) and a higher variability for compositional measurement (plaque attenuation and % of low attenuation plaques), ranging from 4 to 12% for inter-observer variability and 2 to 6% for intra-observer variability.

The radiomic analysis of atherosclerotic plaque may further improve CCTA diagnostic accuracy, given the ability to extrapolate quantitative features of high-risk plaques and stratify plaque risk, with low inter-observer variability. Kolossváry et al. found that 20.6% of radiomic features were significantly different between plaques with and without “napkin-ring sign” and exhibit excellent discriminatory power [55]. When compared with positron emission tomography, intravascular ultrasound and Optical Coherence Tomography plaque assessment, radiomics features on CCTA images showed good-to-excellent diagnostic accuracy in identify vulnerable plaque, surpassing conventional parameters [56].

## Fractional flow reserve

Although high negative predictive value of CCTA in ruling out obstructive CAD [57], the PROMISE trial demonstrated some limitations of CT when used as initial diagnostic strategy, such as the consequent higher rate of ICA not translating into improved clinical outcomes and higher healthcare costs [58].

To improve the ability of CCTA in the identification of flow-limiting coronary stenosis, the calculation of FFR with CT (FFR-CT) is emerging as a robust and valuable tool. The most common method for the calculation of FFR-CT is based on computational fluid dynamics (CFD), a mathematical three-dimensional modeling technique which simulates intra-coronary flow, pressure, and resistance by using semiautomatic contouring and segmentation on CCTA images [59].

**Fig. 4.**
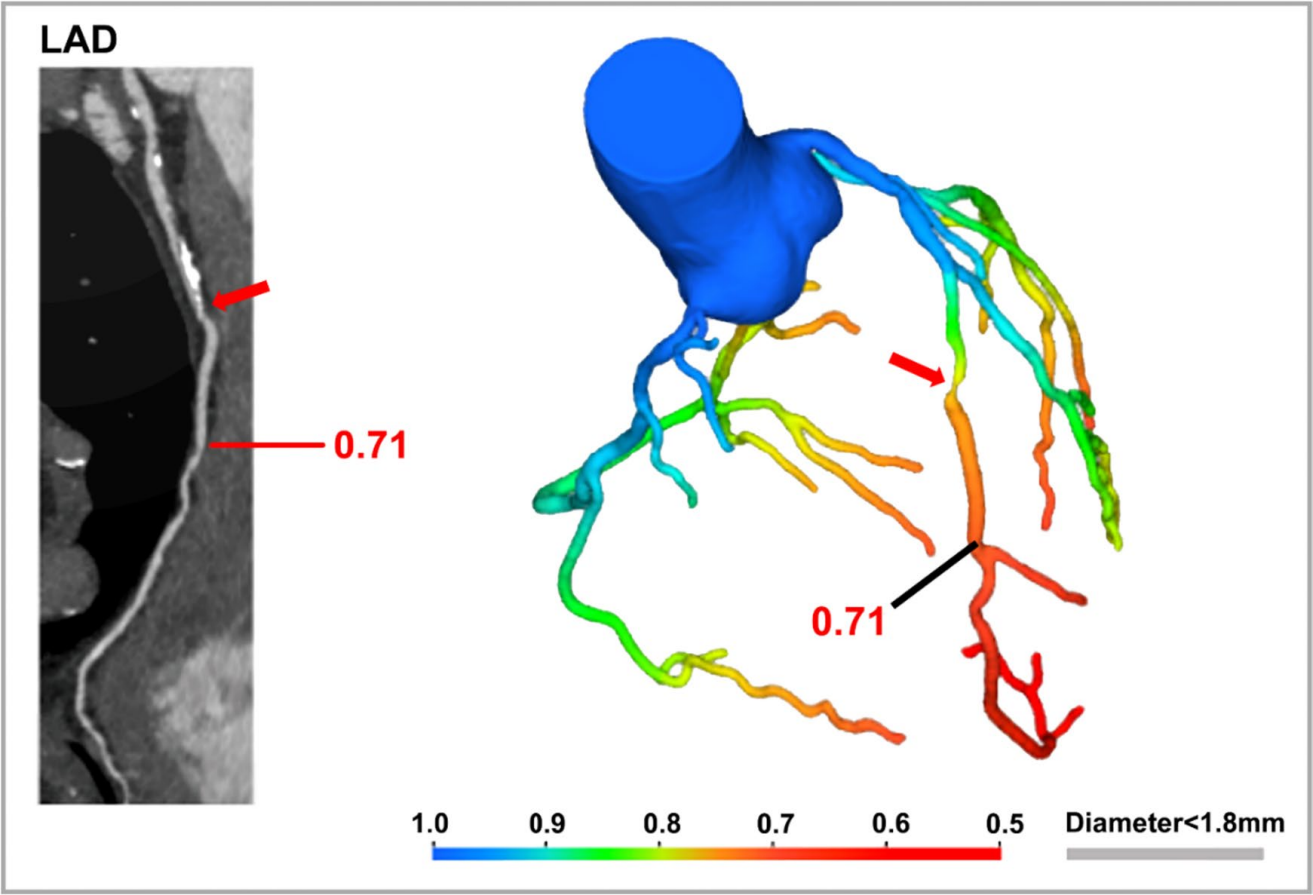
51 years-old man with high cardiovascular risk profile, performing CCTA for recurrent atypical chest pain. Curved planar reformatted images of LAD show a moderate stenosis in middle segment (red arrow). FFR-CT analysis (DeepVessel Image) reveals a significant flow reduction (FFR = 0.71) after the stenosis (red arrow). CCTA, cardiac computed tomography angiography; FFR-CT, fractional flow reserve—computed tomography; LAD, left anterior descending artery

The generated FFR-CT values, based on patient-specific inflow and outflow hemodynamic conditions, are able to predict pressure changes along the course of the vessel. A FFR-CT value greater than 0.8 is normal, values between 0.76 and 0.8 are borderline, and values lower than or equal to 0.75 are abnormal and suggestive for significant stenosis [60] (Fig. 4).

Alongside with CFD techniques, several ML and DL-based methods for FFR-CT have been recently developed [61–64]. The retrospective multicenter MACHINE registry, comparing ML-based and CFD-based FFR-CT, showed no significant differences in the diagnostic performance of ML-approach compared to CFD algorithm [65]. ML and DL-based approaches have the advantage of not requiring transfer of imaging data into the cloud, which increases time consumption. For on-site measurement, the CT-FFR value can be provided to the physician within a day, facilitating prompt decision-making in subsequent steps [66].

In terms of diagnostic accuracy, several trials proved that FFR-CT is a valuable alternative to invasive FFR. In their prospective multicenter study, Bon-Kwon Koo et al. [67] enrolled 103 patients with stable angina who underwent both CCTA with FFR-CT and invasive FFR, demonstrating a good concordance on a per-vessel level (Spearman's rank correlation coefficient of 0.717 and a Pearson's correlation coefficient of 0.678) and no systematic differences at the per-patient level. FFR-CT showed an accuracy of 84% compared to 58.5% of CCTA alone, on a per-vessel analysis; sensitivity and specificity were 87.9% and 83% respectively.

The PLATFORM trial [68, 69] demonstrated that FFR-CT guided strategy was associated with minor rate of MACE at one year compared to the standard-of-care group (6.1% vs. 7.6%, respectively). Moreover, FFR-CT led to a significant reduction in the rate of downstream ICA procedures with median costs lower versus usual care with an invasive strategy (*p* < 0.001).

Recently Fischer et al. [70] sought to explore the role of FFR-CT in the acute setting. They observed that exclusion of hemodynamically significant CAD with FFR-CT in patients with acute chest pain results in a negative predictive value of 100% for excluding MACE at 30 days. Accordingly, the “2021 AHA/ACC/ASE/CHEST/SAEM/SCCT/SCMR Guideline for the Evaluation and Diagnosis of Chest Pain” recommended FFR-CT in intermediate-risk patients with acute chest pain and coronary artery stenosis of 40–90% in a proximal or middle segment on CCTA for diagnosis of vessel-specific ischemia and to guide decision-making regarding the use of percutaneous intervention (PCI) (IIa/B-NR) [71].

While FFR-CT has demonstrated promising results, there are several factors that limit its application in clinical practice, such as the need of optimal quality of CCTA images for adequate post-processing. In some studies, investigating the accuracy of FFR-CT, the percentage of datasets rejected ranged from 11 to 13% [67, 72, 73], reaching 33% in the PROMISE study [58]. Moreover, any inaccuracies in the modeling process can lead to subsequent errors in FFR-CT values; this is particularly valid for small branch vessels which can be left out of modeling, resulting in lack of identification of their stenosis/occlusion by FFR-CT [59].

Despite the aforementioned limitations needs to be taken into account, it should be considered that the introduction of the next-generation hardware may sharply reduce the rejection rate, as demonstrated in the study from Pontone et al. [74], which mostly includes dual-source technology and wide detector scanner, who found a significantly lower rejection rate ranging from 2.9% in the ADVANCE Registry cohort to 8.6% in the clinical cohort. Additionally, they found that temporal resolution, section thickness and heart rate are independent predictors of CCTA scan rejection for FFR-CT analysis, thus suggesting that technological advanced may potentially zeroing the rejection rate by acting on these factors.

## Pericoronary adipose tissue

**Fig. 5:**
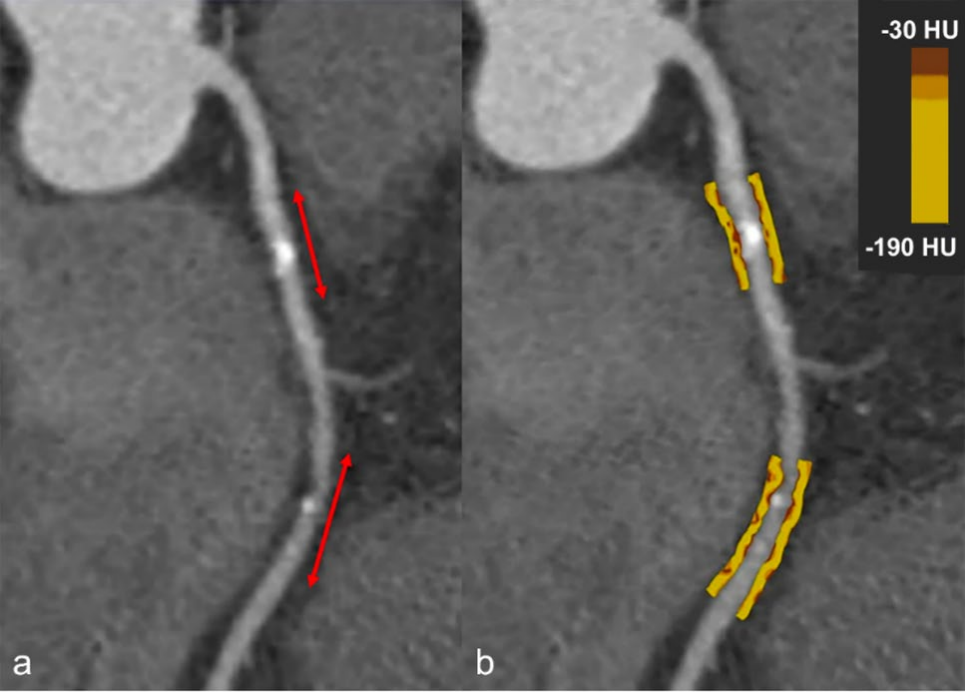
Curved multiplanar reformatted (cMPR) image of LAD shows calcified plaques in proximal and mid segments (**a**). Color-coded cMPR image (**b**) highlights pericoronary adipose tissue (PCAT) attenuation gradients as a metric of vascular inflammation, indicating stable atherosclerotic plaques. LAD Left Anterior Descending artery

It is known that vascular wall inflammation may trigger atherosclerotic plaque instability and risk of rupture, altering lipid accumulation and attenuation in the pericoronary adipose tissue (PCAT) [75]. When inflammatory phenomena occur in the coronary walls, the density of PCAT changes from more negative to less negative values, due to edema and inflammatory cell infiltration (Fig. 5).

Such alterations may be assessed by the perivascular fat attenuation index (FAI) [76], which describes adipocyte lipid content and size, demonstrating excellent sensitivity and specificity for detecting tissue inflammation as assessed by tissue uptake of 18F-fluorodesossyglucose at positron emission tomography [77].

The perivascular FAI is defined as the weighted average attenuation of all voxels containing adipose tissue located within a radial distance from the external vessel wall equal to the diameter of the vessel considered [78]. It can be measured around any segment of the coronary tree, but its original standardization was performed around prespecified segments of the proximal right coronary artery and left anterior descending artery.

Pericoronary FAI is a useful biomarker to detect patients with high levels of vascular inflammation and to identify vulnerable patients at risk for MACEs [79]. As stated by Oikonomu et al. [80], perivascular FAI increases the discriminatory capacity of mortality risk and contributes to the reclassification of current risk stratification models. FAI value is useful in identifying individuals at risk of acute coronary syndrome even in absence of significant coronary stenosis, so as in the identification of vulnerable plaques in patients with known CAD contributing to better clinical and therapeutic management by also providing support in primary and secondary prevention [80].

Going beyond the conventional evaluation of FAI, that only considers the average density measures, the radiomic analysis of PCAT enables the possibility to analyze the attenuation profile considering all the spatial interactions and providing measures of heterogeneity. One of the new radiomic signature of high risk PCAT is the pericoronary Fat Radiomic Profile (FRP). This considers not only the attenuation features included in FAI but also features like fibrosis and vascularity of PCAT. Whereas FAI changes dynamically in response to acute coronary inflammation, FRP captures more permanent structural changes in PCAT and provides additional risk stratification. The combination of FAI and FRP facilitates the development of a more comprehensive individualized cardiac risk profile for each patient [76]. In this regard, Lin et al. recently demonstrated that patients with acute MI show a different PCAT radiomic phenotype as compared to stable CAD patients or healthy controls. PCAT attenuation values were significantly higher in patients with acute MI (− 82.3 ± 5.5 HU) as compared to patients with stable CAD (− 90.6 ± 5.7 HU, *P* < 0.001) and controls with no CAD (− 95.8 ± 6.2 HU, *P* < 0.001) [81].

## Myocardial perfusion imaging

**Fig. 6:**
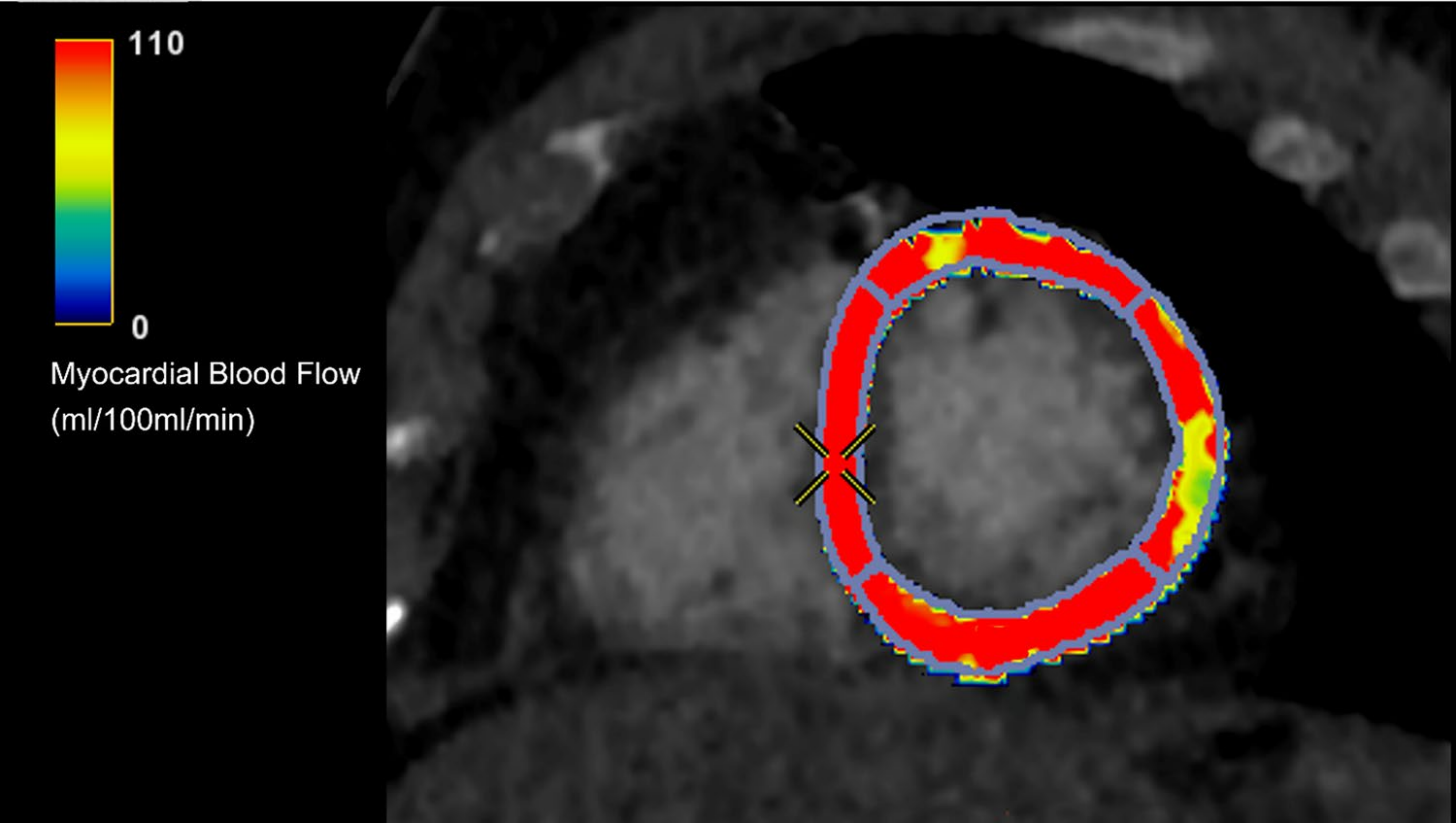
Short axis color-coded parametric map of myocardial blood flow obtained during stress myocardial perfusion CT after injection of Regadenoson showing a perfusion defect in the middle lateral segment of the left ventricle

Stress CT perfusion (CTP) is an emerging imaging technique which combines a pure anatomic evaluation of coronary arteries with functional data. The principle of the technique is based on quantifying the myocardial distribution of the iodinated contrast agent (Fig. 6) as it first passes through the myocardium during pharmacological stress, using vasodilator agents (e.g., Adenosine and Regadenoson) [82] and/or at rest.

Myocardial CTP imaging may involve two types of CT acquisitions: static and dynamic. Static CTP (sCTP) imaging consists in a single snapshot of the myocardial perfusion acquired at a single specific time point during the early first-pass after contrast agent injection. sCTP showed high specificity (68–98%) and sensitivity (50–96%) values, similar to other stress imaging modalities [82]. Nevertheless, sCTP accuracy may be hampered by the possibility to miss the peak of contrast attenuation, leading to false-positive or false-negative results, and limited qualitative/semiquantitative image analysis.

Dynamic CTP (dCTP) imaging may overcome some of these limitations enabling direct measurement of myocardial perfusion. It consists in serial volumetric acquisitions in free-breathing covering the whole heart during contrast injection from the first-pass arterial to the wash-out phases. However, dCTP necessitates of at least a 256–320 slice system scanner or a dual source-CT in order to acquire multiple datasets of images; dCTP also gives a higher radiation dose than sCTP and requires longer breath holding to patients. At the image analysis, several hemodynamic parameters can be extracted, such as the myocardial blood flow (MBF), MBF ratio, and myocardial blood volume (MBV) [83].

Bamberg and colleagues, analyzing the feasibility of dCTP for the detection of significant stenosis using invasive FFR as a reference, and found a significant reduction of MBF and MBV in myocardial segments perfused by stenotic vessels; they also established a cut-off of MBF of 75 mL/100 mL/min for the differentiation between significant and non-significant coronary artery lesions (C statistic, 0.707; *P*, 0.001) [84]. Likewise, Rossi et. al computed an AUC of 0.95 (95% CI 0.92–0.98, *p* < 0.001) for MBF index, on a vessel-territory level, with sensitivity/specificity values of 90/88% in detecting significant stenosis when considering a cut-off value of 78 mL/100 mL/min [85].

dCTP showed greater and additional discriminative effectiveness compared to CCTA alone in various studies [83, 85–88]. In particular, the main diagnostic benefit in detecting significant stenosis of dCTP consists in increasing the specificity value from 61 to 81% as reported in a recent meta-analysis [89]. Moreover, in a multicenter randomized controlled trial, Lubbers et al. [90] demonstrated that a comprehensive CCTA protocol with myocardial perfusion led to fewer additional noninvasive testing and shorter diagnostic pathways.

Myocardial perfusion may also ameliorate prognostic prediction, as the summed stress score, determined by normalizing MBF using CTP, is a better predictor of MACE then coronary stenosis at CCTA, with a hazard ratio of 5.7 (95% confidence interval: 1.9–16.9; *p* = 0.002) [91]. MBF extracted from dCTP is also highly accurate in the assessment of microvascular obstruction [92], which is known to be a predictor of MACE in patients with myocardial infarction and preserved ejection fraction [93].

Finally, given that iodine is delivered to myocardial tissue by blood flow supply, recent studies suggest iodine distribution maps by DECT as a marker of myocardial perfusion [94, 95]. Introducing iodine perfusion maps increases the diagnostic accuracy of CCTA scans compared to cardiac magnetic resonance (CMR), single-photon computed tomography and ICA [96].

## Late iodine enhancement

**Fig. 7:**
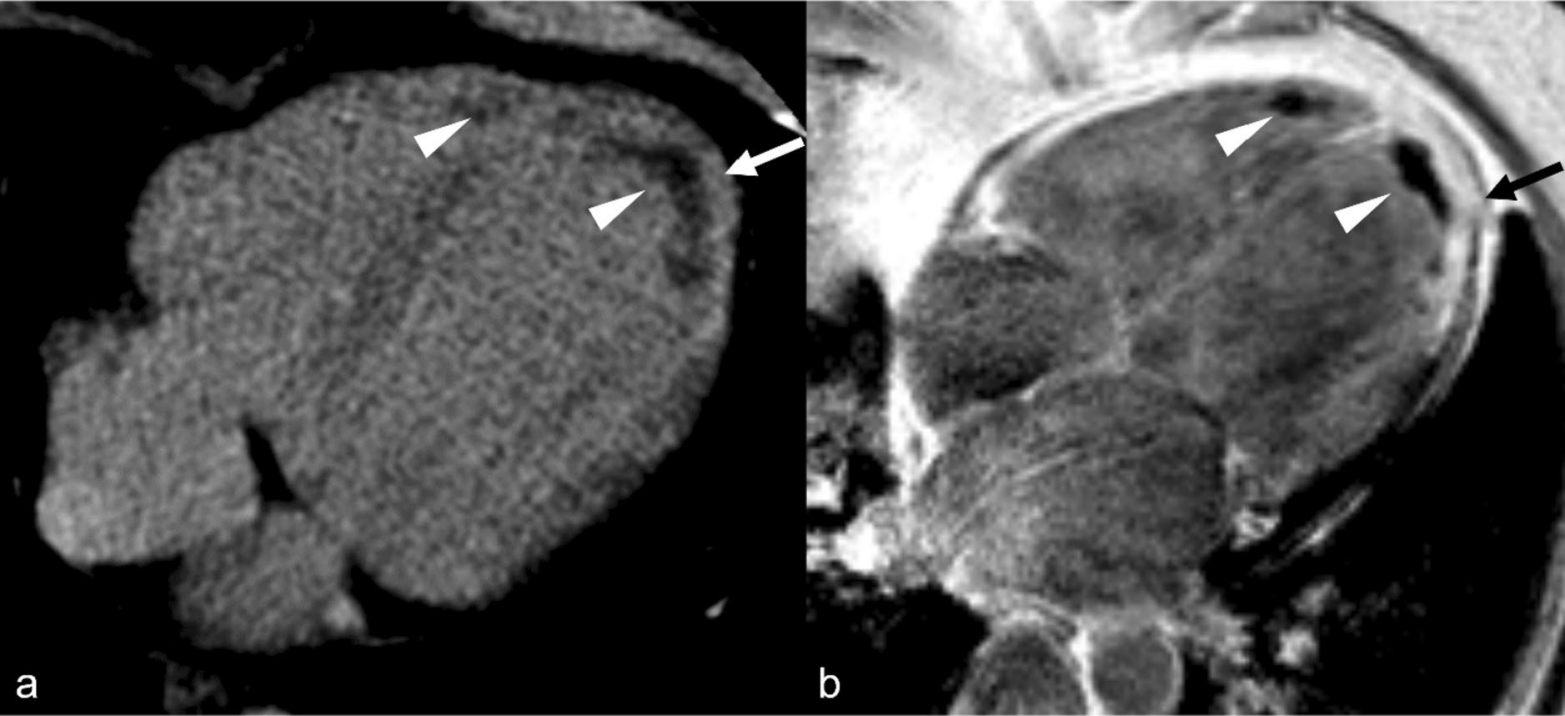
Late iodine enhanced (LIE) CT image reconstructed in four chamber view (**a**) and late gadolinium enhanced (LGE) image acquired on the corresponding plane during a cardiac-MR (**b**) in a patient with an acute myocardial infarction of the left circumflex artery territory. In both images is evident the contrast enhancement of the infarcted myocardium (white and black arrows) and the thrombi adherent to the apex in both ventricles (white arrowheads)

Imaging of myocardial fibrosis (MF) is based on contrast agent accumulating in myocardial tissue areas which demonstrate an expansion of extracellular matrix or in the intracellular space of necrotic myocytes [97]. Late gadolinium enhancement (LGE) imaging by CMR represents the reference standard to assess MF in vivo, but several contraindications to CMR exist together with the long image acquisition times, which limits its use. The evaluation of MF scars has also been developed with CT, based on the extracellular properties of iodine CM and the visualization of hyper-enhanced areas due to iodine accumulation [98], showing a diagnostic accuracy of 88–95% as compared to CMR-LGE [99] and a diagnostic accuracy of 90%, with 53% sensitivity and 98% specificity, if compared to histological examination [100] (Fig. 7).

Several protocols exist regarding the injection of CM and the timing of late-phase acquisition: the majority of the authors report a single-bolus administration technique, so the late phase is acquired after the standard dose of CM injected for CCTA, with no further amount of CM; in other cases, a bolus-continuous protocol is performed, with an additional continuous infusion of CM (30–90 mL at 0.1–0.3 mL/s) after the CCTA scan. The optimal timing for the late phase scan ranges between 5 and 15 min, with the best results reported between 10 and 15 min using the bolus-continuous protocol [98].

Because of poor contrast resolution in the delayed-phase CT scan, especially for subepicardial scars, the assessment of MF can be challenging [99]. Values of diagnostic accuracy vary according to the image reconstructions algorithms that are applied, ranging from a sensitivity and specificity of 56% and 93% (with filtered back projection) to 80% and 91% (with knowledge based iterative model reconstruction), respectively [101]; and sensitivity decreases with the increasing in tube voltage kVp reconstructions, ranging from 98% at 100 kVp, to 28% at 140 kVp [102]. In this regard, the use of low tube voltages (80–100 kVp) and specific denoising reconstruction algorithms can improve contrast-to-noise ratio and reduce the radiation dose to the patients [103].

DECT and PCD-CT technology allow for spectral evaluation and virtual monoenergetic images (VMI) reconstruction at lower KeV, resulting in improved contrast-to-noise ratio for LIE evaluation [100]. Spectral CT demonstrated a sensitivity of 82% and a specificity of 99% among a population of patients with CMR-proven acute myocarditis [104]. Using iodine-density imaging, sensitivity was 97.1% and specificity 88.9% in patients with heart failure, with the highest diagnostic accuracy obtained for 40-keV VMI reconstructions (90.8%) [105]. LIE can also be used to investigate acute chest pain patients admitted to emergency department with troponin increase [106]: with this approach, patients are scanned in the angiographic phase to rule out (obstructive coronary artery disease, acute aortic syndromes and pulmonary embolism) and in the late phase for myocardial tissue characterization. Other applications include the assessment of myocardial scars as a substrate for ventricular arrhythmias prior to radiofrequency catheter ablation procedures [107].

## Extracellular volume fraction

Extracellular volume fraction (ECV) assessed using T1 mapping sequences by CMR (ECV_CMR_) has emerged as valuable surrogate marker of interstitial fibrosis, calculated by the amount of gadolinium distributed in the myocardium in the equilibrium phase [108]. ECV may be assessed also with CT (ECV_CT_) with similar approach [109], overcoming the aforementioned contraindications related to CMR [110]. Recent studies that compared ECV_CMR_ with ECV_CT_ among patients with amyloidosis, aortic stenosis, pulmonary hypertension, or dilated cardiomyopathy achieved correlation coefficients ranging from 0.73 to 0.91 [111, 112], whereas the correlation between collagen volume fraction at histology and ECV_CT_ values in a population with severe aortic stenosis was 0.71 (*p* = 0.0007) [109].

**Fig. 8:**
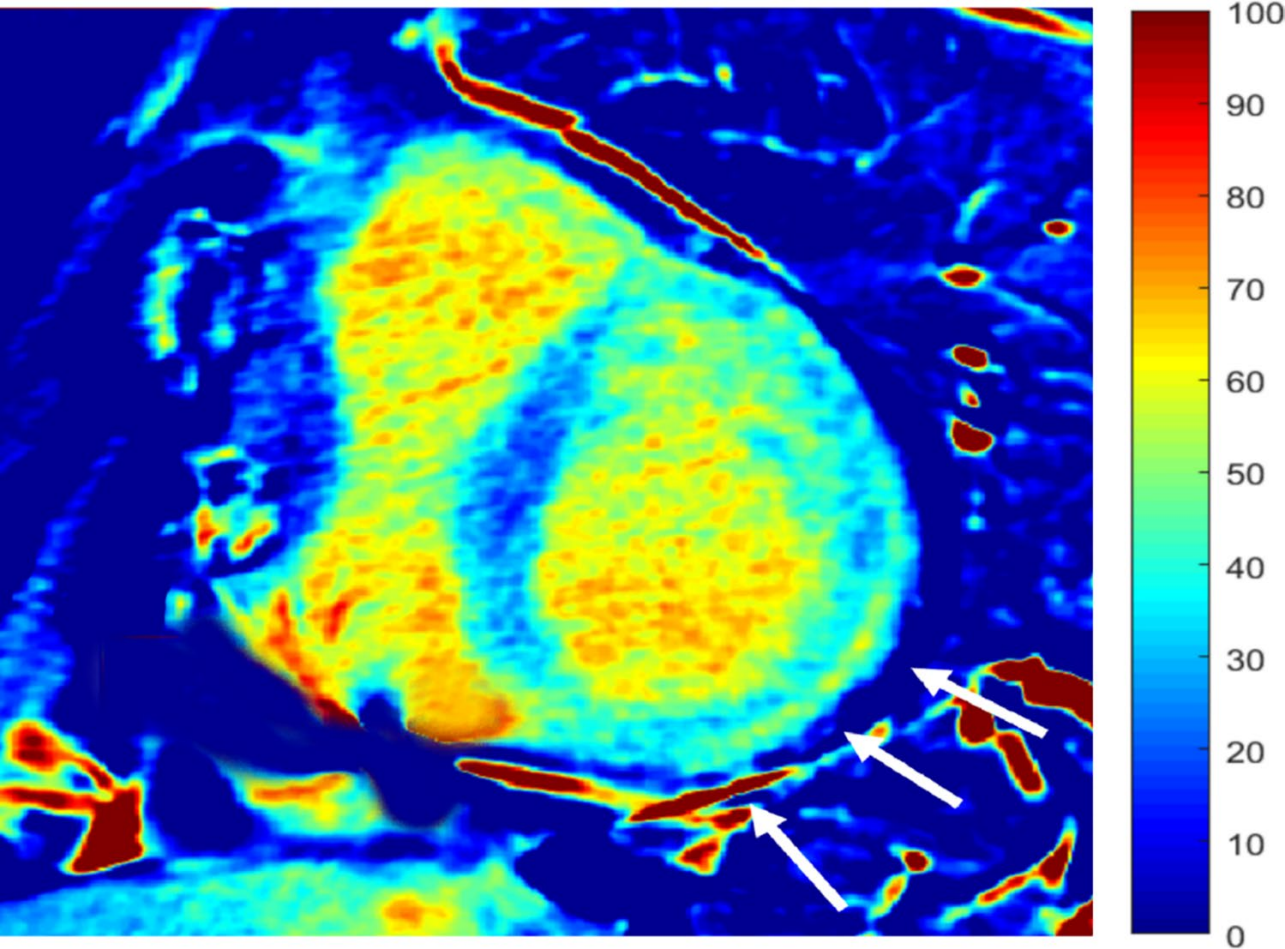
CT-derived extracellular volume fraction (ECV_CT_) map on a mid-ventricular short axis slice acquired in a 43-years-old woman with history of ventricular tachycardia and previous ICD implantation. The map shows a diffuse increase of ECV in the lateral wall (white arrows) associated to a thin subepicardial rim of focally increased ECV compatible with scarring fibrosis. The patient was diagnosed with chronic myocarditis. *ICD: implantable cardioverter device*

ECV measurements can be performed with single-energy acquisitions, with DECT or PCD-CT [113] (Fig. 8).

In the former method, ECV_CT_ is derived by combining the differences in attenuation in myocardial tissue and blood pool, between the delayed scan and the non-enhanced scan. However, this technique can be associated with misregistration artifacts (non-matching images between baseline and late phases) and a greater radiation dose to the patients [114, 115]. With DECT or PCD-CT, ECV_CT_ is quantify by measuring the iodine concentration in myocardium and blood pool in the delayed-phase scan only, based on spectral decomposition of the obtained multi-energy datasets [110]. The acquisition time for the delayed scan phases is not still unanimously shared, ranging from 5 to 12 min [114, 116]; CM injection technique could be based on single or double boli (fixed or proportional to patient’s body weight [117–119]) or with slow intravenous infusion [109, 115].

ECV_CT_ represents a promising biomarker, that could support the management of cardiac diseases associated to the development of MF, improving the prediction of MACE [120], mortality [121] or progression to heart failure [122, 123] and addressing specific therapies.

## Epicardial adipose tissue

**Fig. 9:**
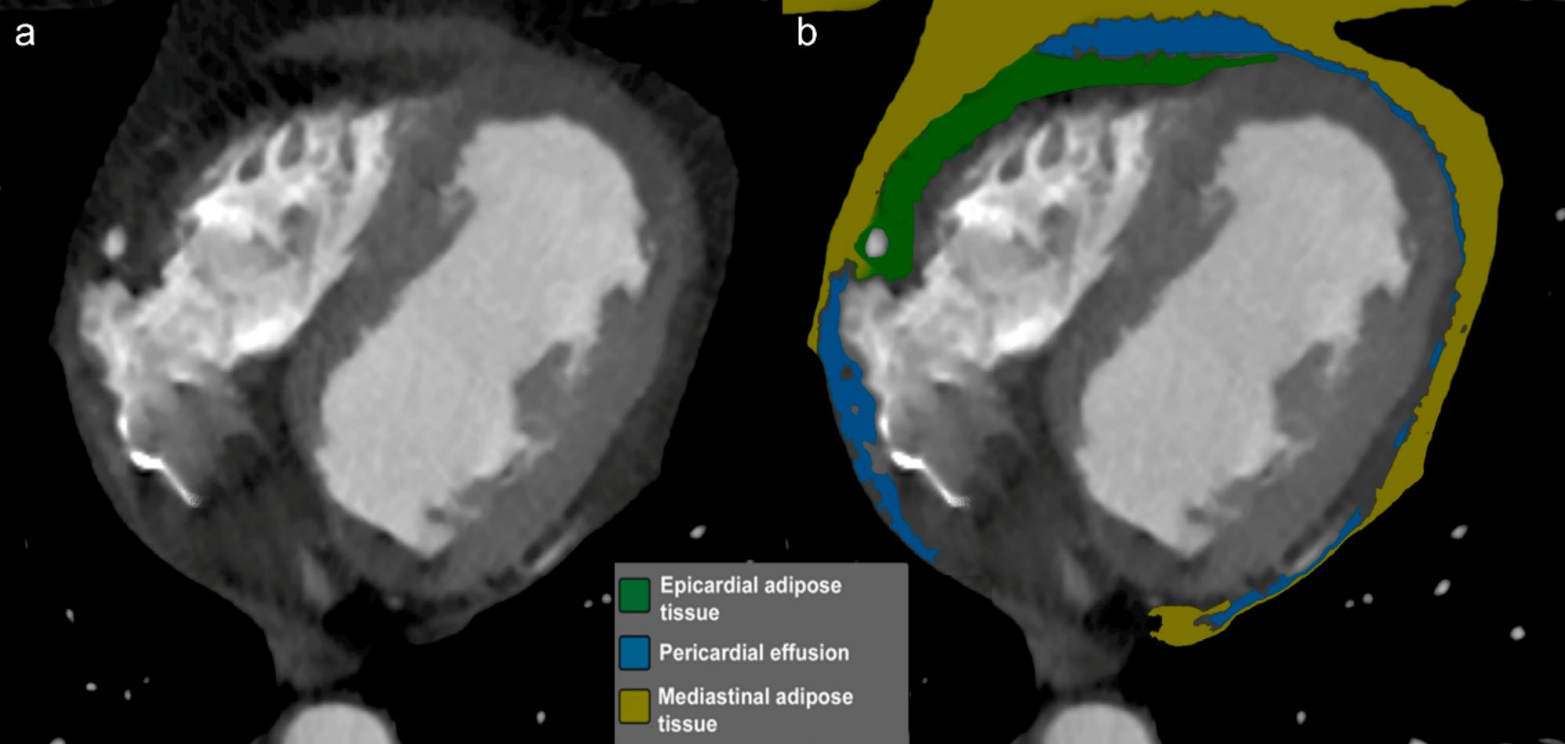
Axial CT (**a**) and corresponding color-coded map (**b**) showing different pericardial (yellow) and epicardial (green) adipose tissue compartments

Epicardial adipose tissue (EAT) typically appears as a hypodense layer lying between the myocardial wall and the visceral pericardium, with a density ranging from − 190 to − 30 HU containing the coronary vessels and their principal branches [124–126] (Fig. 9).

In the last few decades, growing attention have been focused on EAT as a biomarker of CV risk, as an increased EAT volume has shown a strong association with CV pathologies, including CAD and ischemic heart disease [127, 128]. Specifically, Mancio et al. [125] showed that EAT volume was associated with coronary stenosis, myocardial ischemia, and MACE, irrespective from CV risk factors.

Increased EAT volume has also proven to be a strong predictor for the risk of atrial fibrillation [129], regardless of other risk factors, including left atrium diameter [130]. In patients with heart failure with preserved ejection fraction, greater EAT deposition have been associated with higher body mass index (BMI), cardiac structural changes, and proteomic markers linked to general obesity, systemic inflammation, insulin resistance, endothelial dysfunction, and dyslipidemia [131]. EAT volume quantification is feasible on both non-contrast cardiac-CT and CCTA image data sets, even though the presence of contrast media (CM) may lead to an underestimation of EAT volume [132].

Beyond the volumetric quantification, the analysis of EAT radiodensity may also serve as an imaging biomarker, as it may reflect inflammation or metabolic activity of EAT [133]. Franssens et al. [134] found a significant association between low CT attenuation of EAT and a higher amount of CAC in men with higher CV risk or overt CV pathology, regardless of EAT volume and BMI. Accordingly, serum levels of plaque inflammatory markers, coronary calcification, and MACE were all linked with lower EAT density in a study from Goeller and colleagues [135]. Finally, a rise in EAT radiodensity is also associated with Tako-Tsubo syndrome and myocardial infarction with non-obstructive coronary artery (MINOCA) [136].

Besides manual quantification, which may be highly time-consuming, in the last few years methods allowing semi-automatic [135, 137, 138] and automatic segmentation [139, 140], including DL approaches [141, 142], have been investigated. Specifically, the adoption of fully automated segmentation techniques may provide the benefit of high reproducibility for EAT volumes calculation with quicker segmentation times, optimizing EAT quantification for clinical use [141].

## Future perspectives

The introduction of radiomics into the medical field represents a chance to explore quantitative data through the extraction of multiple image-based features that are imperceptible to the naked eye [143]. The initial experience using radiomics analysis in cardiac CT imaging [144–149] indicate that this technique may help in identifying vulnerable plaques, improve cardiac risk stratification and open new frontiers for personalized cardiovascular medicine [150]. On the other hand, research into potential applications of AI in diagnostic imaging has gained growing interest in the last decade, and cardiac imaging was not an exception. With the rise of commercially available or open-source AI algorithms, the workflow of postprocessing analysis and interpretation of CCTA imaging datasets is changing dramatically. The optimization and speeding up of procedures guaranteed by AI tools, are expanding the role of CCTA for risk stratification as well as for patient treatment planning and management [151].

The progressive validation of new CT imaging biomarkers will expand the role of Cardiac CT in the next few years, moving beyond the simple assessment of stenosis to risk stratification and characterization of tissue alterations (Table 1). The automatic extraction of various quantitative parameters from cardiac CT imaging dataset could open a new era, where several features of vulnerability, functional parameters and markers of tissue changes will be provided, enabling deeper phenotyping of the disease and addressing to personalized therapeutic approach.

**Table 1:** Summary of CT imaging biomarkers

Biomarker	Acquisition Technique	Contrast administration (Phase)	Definition	Utility	Advantages	Limitations
Calcium score (Agatston Score, calcium volume and mass)	ECG-gated unenhanced scan	No	Measurement of the amount of calcium in the coronary arteries wall	Estimation of CAD burden Improvement of risk stratification for MI or other MACEs (including in asymptomatic patients)	Low radiation dose No use of contrast agents Easy post-processing	No standardized extraction of calcium scores values from CCTA images Inability to discriminate between focal and scattered calcified plaques Inability to detect non-calcified plaques
Segment Involvement Score (SIS)	ECG-gated contrast enhanced scan	Yes (arterial)	Total number of coronary segments with atherosclerotic plaque		Semi-quantitative assessment Considers both calcified and non-calcified plaques Indicator of CAD also at early stages	Time-consuming analysis Poor discrimination between patients with or without flow-limiting stenosis and/or requiring revascularization
Segment Stenosis Score (SSS)	ECG-gated contrast enhanced scan	Yes (arterial)	Summation of the extent scores of all 16 individual segments Score is calculated as: 0 = no plaque; 1 = < 50%; 2 = 50–69%; 3 = > 70%			
Leaman score	ECG-gated contrast enhanced scan	Yes (arterial)	A score based on 3 weighted factors of coronary plaques: (1) localization; (2) type of plaque; (3) degree of stenosis (< 50 ≥ % stenosis) for each segment. The total score represents the sum of partial score for each segment			
Leiden score	ECG-gated contrast enhanced scan	Yes (arterial)	A score based on: plaque location (0–6 points); the severity of the stenosis (1–1.4 points) and the composition (1–1.3 points) of coronary plaques. The total represents the addition of each individual segment scores, obtained by the multiplication of these three factors			
CAD-RADS 2.0	ECG-gated contrast enhanced scan	Yes (arterial)	The score is mainly based on the stenosis of most severe degree. The updated classification includes: plaque burden; ischemia evaluation (integration of CT-FFR or CTP); modifiers like coronary stents, high-risk plaque features, ischemia test results, and plaque severity, based on CAC, SIS, and Visual scoring	It offers practical recommendation for clinical management of the patients	It combines information on the degree of stenosis, atherosclerotic burden and plaque characteristics	Simplistic scoring system Time Consuming It is particularly conditioned by the degree of the major stenosis Low reproducibility of high risk plaque features
Fractional Flow Reserve (FFR-CT)	ECG-gated contrast enhanced scan	Yes (arterial)	Computational fluid dynamic-based estimation of blood flow through vessels affected by coronary stenosis using a CT anatomical 3D model	Estimation of coronary flow Detection of flow-limiting stenosis	Valuable alternative to invasive FFR High Negative Predictive Value Improving selection of patients candidate to ICA	Limited accessibility as a result of the pay-per-service approach Single provider approved for clinical use
Pericoronary Adipose Tissue (FAI; FRP)	ECG-gated (contrast enhanced or unenhanced) scan	Not necessary	FAI: average weighted attenuation of adipose tissue-containing voxels surrounding the coronary FRP: radiomic mapping of the pericoronary space based on various form, attenuation, and texture-related characteristics	Improvement of risk stratification for MI or other MACEs Information on metabolic activity of PCAT (fat browning)	Useful in assessment of vulnerable plaque not associated with significant stenosis Possibility of application of radiomic tools	Time consuming analysis Few available dedicated software Influence of acute coronary inflammation on FAI
Static myocardial perfusion	ECG-gated contrast enhanced scan	Yes (arterial)	Myocardial iodine distribution acquired at a single specific time point during the early arterial phase	Detection of coronary artery lesion-specific ischemia	Combined CT perfusion and CCTA in a single image dataset Short exam duration (compared to other cardiac functional tests) Rapid analysis Lower radiation dose	Qualitative/semiquantitative analysis (dedicated software) Need for wide coverage CT scanner High-dependance on acquisition time Non-standardized evaluation of the iodine distribution maps Use of stressor agents
Dynamic myocardial perfusion (MBF and MBV)	Serial ECG-gated contrast enhanced scans	Yes (from arterial to wash-out)	Myocardial iodine distribution acquired over consecutive volumetric scans during passage of contrast agent	Detection of coronary artery lesion-specific ischemia	Direct measurement of myocardial perfusion throughout quantitative analysis Less influence of acquisition time Short exam duration (compared to other cardiac functional tests)	Increased radiation and contrast dose Longer breath-hold Spatial misalignment resulting from table movements Need for wide coverage CT scanner Time-consuming analysis (dedicated software) Use of stressor agents
Late Iodine Enhancement	ECG-gated contrast enhanced scan	Yes (late, 5–12 min after injection)	Myocardial areas with a focal increase of attenuation compared with remote myocardium	Detection of myocardial fibrosis	Feasible also in patients with CMR contraindications (implantable devices, claustrophobia) Applicable in setting of emergency (e.g., acute chest pain patients with troponin increase) Shorter exam duration as compared to CMR	Lower contrast to noise ratio than CMR Increased radiation exposure as compared to CCTA alone
Extracellular Volume Fraction (ECV_CT_)	ECG-gated unenhanced + contrast enhanced scan	Yes (late, 5–12 min after injection)	Extracellular volume fraction mapping using iodine distribution during equilibrium phase	Quantification of extracellular matrix expansion	Feasible also in patients with CMR contraindications (implantable devices, claustrophobia) Shorter exam duration as compared to CMR Only late phase scan if spectral imaging is used	Preferable latest generation scanner for dose control and imaging optimization Misregistration artifacts Increased radiation exposure as compared to CCTA alone Need for dedicated software
Epicardial Fat Volume/Density	ECG-gated (contrast enhanced or unenhanced) scan	Not necessary	Quantification of epicardial adipose tissue volume and density	Improvement of risk stratification for MI or other MACEs Information on metabolic activity of EAT (fat browning)	Providing information on metabolic and inflammatory status	Time consuming analysis Few available dedicated software

CMR, cardiac magnetic resonance; CCTA, coronary computed tomography angiography; CT, computed tomography; FFR-CT, CT-derived fractional flow reserve; CV, cardiovascular; FAI, Fat Attenuation Index; FRP, Fat Radiomic Profile; ICA, invasive coronary angiography; MACE, major adverse cardiac event; MBF, Myocardial Blood Flow; MBV, myocardial blood volume; PCAT, pericoronary adipose tissue; ECV_CT_, CT Extracellular Volume

## Conclusion

The role of CCTA has progressively grown from the mere detection of obstructive CAD, by the anatomical assessment of coronary stenosis, to an examination that enables the extrapolation of multiple parameters, useful for a more accurate evaluation of cardiovascular risk and the hemodynamic effect of stenosis. In addition, new applications of CT (e.g.,, LIE, ECV_CT_) are expanding its domain to the characterization of myocardial damage [152] and improving prognostic stratification, moving toward an increasingly comprehensive examination.

## Abbreviations

AI: Artificial intelligence
AS: Agatston score
BMI: Body mass index
CAC: Coronary artery calcium
CAD: Coronary artery disease
CCTA: Cardiac computed tomography angiography
CFD: Computational flow dynamics
CM: Contrast media
CMR: Cardiac magnetic resonance
CT: Computed tomography
CT-LeSc: CT-adapted Leaman score
dCTP: Dynamic CT perfusion
sCTP: Static CT perfusion
CV: Cardiovascular
DECT: Dual energy computed tomography
DL: Deep learning
EAT: Epicardial adipose tissue
ECV: Extracellular volume
EMB: Endomyocardial biopsy
FAI: Fat attenuation index
FFR: Fractional flow reserve
FRP: Fat radiomic profile
HR: Hazard ratio
HU: Hounsfield units
ICA: Invasive coronary angiography
IHD: Ischemic heart disease
LGE: Late gadolinium enhancement
LIE: Late iodine enhancement
MACE: Major adverse cardiac events
MBF: Myocardial blood flow
MF: Myocardial fibrosis
MI: Myocardial infarction
MINOCA: Myocardial infarction with non-obstructive coronary artery
ML: Machine learning
PCAT: Pericoronary adipose tissue
PCD-CT: Photon-counting detector computed tomography
RI: Remodeling index
SIS: Segment involvement score
SSS: Segment stenosis score
VMI: Virtual monoenergetic images
